# LGBTQ+ experiences of accessing NHS adult mental health services during COVID-19 in an area of North West England: a qualitative interview study

**DOI:** 10.1186/s12913-026-14014-2

**Published:** 2026-02-07

**Authors:** Hayley J. Lowther-Payne, Neil Caton, Keith Holt, Adelaide Beckwith, Anastasia Ushakova, Fiona Lobban

**Affiliations:** 1 Lancaster Medical School, Faculty of Health and Medicine, Health Innovation One, Lancaster University, Sir John Fisher Drive, Lancaster, Lancashire LA1 4AT UK; 2 Division of Health Research, Faculty of Health and Medicine, Lancaster University, Health Innovation Campus, Sir John Fisher Drive, Lancaster, Lancashire LA1 4AT UK; 3 Department of Health Services Research, National Institute of Health and Care Research (NIHR) Applied Research Collaboration North West Coast (ARC NWC), University of Liverpool, Waterhouse Building, Liverpool, L69 3GL UK; 4 Centre for Applied Health Research, University of Salford, Salford, Greater Manchester M5 4WT UK

**Keywords:** LGBTQ+, COVID-19, Healthcare access, Inequalities, Mental health services, England, Qualitative

## Abstract

**Background:**

Lesbian, gay, bisexual, transgender, and queer (LGBTQ+) people have a higher prevalence of mental health conditions than heterosexual and cisgender people, and report poor experiences of accessing mental health services. The COVID-19 pandemic may have had detrimental impacts on socially disadvantaged groups, such as LGBTQ+ people, and their mental healthcare access. This study aimed to understand LGBTQ+ experiences of mental health and accessing mental health services during the COVID-19 pandemic.

**Methods:**

Topic-guided qualitative interviews were conducted with twelve LGBTQ+ people who accessed or tried to access NHS mental health services in an area of North West England between March 2020 and February 2022. Levesque’s Conceptual Framework for Healthcare Access was used as a framework to code the qualitative data. Reflexive thematic analysis was adopted to analyse the qualitative data from a critical realist perspective. Researchers with lived experience of being LGBTQ+ and accessing mental health services were embedded as part of the research team.

**Results:**

Eight themes across five domains of the framework were identified from the qualitative data. LGBTQ+ participants shared the negative impacts the pandemic had on their mental health (e.g., worsening existing mental health conditions, experiences of isolation, a loss of social connectedness with the LGBTQ+ community). LGBTQ+ participants experienced significant challenges accessing mental health services during COVID-19, associated with experiences of stigma and discrimination, concerns about disclosing their LGBTQ+ identity, living in unsupportive environments, and being unable to access mental health support remotely. Where positive experiences were identified, these highlighted important opportunities for change.

**Conclusions:**

This study suggests that LGBTQ+ people may have been adversely impacted by the COVID-19 pandemic, with an increased risk of mental ill health and isolation, and poor experiences of accessing mental health services. Opportunities to maximise protective factors and improve timely access to mental health support are needed to mitigate these effects for LGBTQ+ people in the future. Recommendations to improve LGBTQ+ service inclusivity include implementing self-referral options, being more visibly LGBTQ+ inclusive, and improving staff knowledge and training on supporting LGBTQ+ service users.

**Supplementary Information:**

The online version contains supplementary material available at 10.1186/s12913-026-14014-2.

## Background

Lesbian, gay, bisexual, transgender, and queer (LGBTQ+) people have a higher prevalence of mental health conditions, when compared to heterosexual people [1, 2], and cisgender people (those whose gender is the same as the sex they were assigned at birth) [3]. Whilst one in five adults in England report having a common mental health condition [4], over half of LGBTQ+ people report they have experienced depression or anxiety [5]. To date, these inequalities have largely been attributed to “minority stress” [6], whereby exposure to stress-inducing experiences of stigma and discrimination (e.g., homophobia, transphobia) can have a detrimental impact on mental health. Sexual and gender minority groups experience minority stressors, such as experiences of violence and abuse [7], a lack of social support [7, 8], a lack of social safety [9], and societal cis-heteronormativity [10], which contribute to disproportionately higher levels of mental ill health. These disparities are also maintained through ineffective mental healthcare that is not responsive and inclusive to the needs of diverse population groups [11]. Despite being at a higher risk of mental health conditions, LGBTQ+ population groups are under-researched when considering access to and experience of mental health services in the United Kingdom (UK).

Fears about encountering stigma and discrimination when accessing mental health services [12], the potential pathologisation of their LGBTQ+ identity [13], or the view that healthcare professionals lack knowledge to effectively support LGBTQ+ people [1], may affect those who identify as LGBTQ+ and their ability to “seek”, “reach”, or “engage” with mental health services. Gender minorities specifically experience barriers to accessing gender-affirming healthcare (care that is delivered to support and affirm an individual’s gender identity); in that it requires a considerable amount of agency to navigate, involves long delays and waiting times, and has been associated with unnecessary referrals to mental health services [14]. Disclosure of sexual orientation and gender identity when accessing healthcare has been associated with poor responses from healthcare professionals, such as refusal of care, dismissal, and discrimination [15]. On the other hand, when an individual’s sexual orientation and/or gender identity are not asked for or not disclosed, psychological treatment and therapeutic relationships may be less effective and and associated with poorer outcomes [12]. Mental healthcare is designed and delivered to meet the needs of a cis-heteronormative society [11], and as such LGBTQ+ people often report poor experiences of accessing mental health services [1].

The effects of the COVID-19 pandemic were not experienced equally in society, with inequalities in mental health identified for various population groups (e.g., young people, people from lower income backgrounds, ethnic minorities) [16, 17]. Evidence suggests that the health and well-being of LGBTQ+ population groups was disproportionately affected by the COVID-19 pandemic [18], experiencing unique challenges such as increased exposure to discrimination, isolation, and loss of access to supportive spaces and affirmative care [19, 20]. Bécares and Kneale [21] analysed survey data from two waves of the UK Millenium Cohort Study, identifying significant inequalities in social support and self-rated physical and mental health among sexual minority young adults compared to heterosexual adults during COVID-19, Using a cross-sectional survey during the first UK lockdown, Kneale and Bécares [22] found that LGBTQ+ respondents had high levels of perceived stress and depressive symptoms when compared with standardised thresholds, which was in part explained by experiences of sexuality and gender-based discrimination during the pandemic. A secondary analysis of this survey highlighted how the COVID-19 pandemic exacerbated the “psychosocial hostility” experienced by LGBTQ+ people, in particular for transgender and non-binary individuals, resulting in a further determinantal impact on their mental health [23]. The LGBT Foundation’s “Hidden Figures” report in 2020 reported that 42% of their LGBTQ+ survey sample (*n* = 555) wanted to access support for their mental health during the first COVID-19 lockdown [24], a disproportionately higher proportion than one in six adults who accessed mental health treatment in the general population [4, 25]. Beyond the UK, evidence indicates that the COVID-19 pandemic had negative psychological impacts on LGBTQ+ people globally [26–28].

Whilst there is evidence to highlight the disproportionate effects of COVID-19 on LGBTQ+ mental health [19, 20], there is limited research exploring the experiences of LGBTQ+ people accessing mental health services during the pandemic in the UK. To what extent the pandemic and associated restrictions impacted the lives of LGBTQ+ people and their healthcare access specifically has been largely absent from COVID-19 research, and sexual orientation and gender identity data were also omitted from any of the public health surveillance [18]. Intensifying hostile attitudes towards LGBTQ+ people and UK political decisions to abandon the 2018 LGBT Action Plan [29] and to not mandate the 2017 NHS Sexual Orientation Monitoring Information Standard [30] during the pandemic have led to a lack of focus on improving the health and well-being of LGBTQ+ people and continue to perpetuate inequalities, which remain understudied. It is important to ascertain the healthcare experiences of underserved population groups during COVID-19 to inform future actions to improve the inclusivity and equity of mental health services and to mitigate against disproportionate effects if significant disruptions were to happen to services again. This qualitative interview study aimed to capture the experiences of people who identify as LGBTQ+ who accessed or tried to access adult NHS mental health services during the COVID-19 pandemic, to address the following research questions:

1. How did people who identify as LGBTQ+ experience the impacts of the COVID-19 pandemic on their mental health and their access to mental health services?
2. How were these experiences impacted by their LGBTQ+ identity?

## Methods

### Study design and setting

This was a qualitative topic-guided interview study conducted between September 2023 and April 2024. This study is reported in accordance with the Consolidated Criteria for Reporting Qualitative Research (COREQ) checklist [31]. This study was conducted in Lancashire and South Cumbria, a large geographical area in the North West of England with a population of approximately 1.8 million people [32]. There is considerable variation in the health and well-being across the region, with significant disparities in life expectancy [33], prevalence of mental and physical health conditions [34, 35], and concentration of social deprivation [36]. NHS mental health services in Lancashire and South Cumbria are under-funded and under significant pressure due to the high rates of adults in contact with services compared with the national average [37], which has been compounded by the disproportionate impacts of the COVID-19 pandemic (e.g., higher rates of mortality and hospital pressures, longer periods of time under restrictions, larger reductions in self-reported mental well-being) [38]. According to the Census 2021, in Lancashire and South Cumbria, 40,035 (2.9%) people identify as a sexual minority [39], and 5,608 (0.4%) people identify as a gender minority [40]. These proportions are however likely to be an underestimation, with 6.9% not answering the sexual orientation question and 5.5% not answering the gender identity question.

### Theoretical framework

Levesque’s Conceptual Framework for Healthcare Access [41] was drawn upon in this study to conceptualise a definition of “access”. According to the framework, healthcare access is viewed as a multi-dimensional concept associated with healthcare systems and their approachability, acceptability, availability, affordability, and appropriateness, and with individuals and their ability to perceive, seek, reach, pay, and engage with healthcare services [41]. Whilst other frameworks were considered for this study (e.g., Dixon-Woods’ Candidacy framework [42]), Levesque’s framework offered a lens through which dimensions of access related to the healthcare system, such as the availability of services during COVID-19, and the appropriateness of remote delivery during COVID-19, along with dimensions of access related to LGBTQ+ population groups’ abilities to seek, reach, and engage with services during COVID-19, could be explored.

### Study population and recruitment

Ethical approval was obtained for this study in June 2023 from Lancaster University’s Faculty of Health and Medicine Research Ethics Committee (FHM-2023-3639-RECR-1). Convenience sampling was used to recruit participants who were aged 18 years and over, identified as LGBTQ+, and had accessed or tried to access NHS adult mental health services in Lancashire and South Cumbria between March 2020 and February 2022. The configuration of NHS mental health services across England is highly varied and so limiting to this geographical area meant that participant experiences could be compared more easily. Participants were recruited via electronic adverts shared on social media and with LGBTQ+ organisations and networks across Lancashire and South Cumbria, and via paper adverts placed in local spaces, such as community centres and cafes, where third sector organisations offer LGBTQ+ support sessions. Potential participants were asked to contact the researcher via email to check their eligibility, provide a copy of the participant information sheet and a consent form, answer any questions, and arrange a suitable time for the interview. Prior to the interview, an online monitoring form was completed by participants in Qualtrics [43] to capture demographic information.

### Data collection

One-to-one interviews were conducted by a researcher (HL) using videoconferencing software, Microsoft Teams. Due to the sensitive nature of the topic, interviews were considered more appropriate to facilitate an open and supportive discussion with participants than alternative methods (e.g., focus groups). Written and verbal consent was gained from all participants prior to the interview. A topic guide was used to guide the conversation (Figure S1 in Additional file 1), the development of which was informed by Levesque’s Conceptual Framework for Healthcare Access [41]. The topic guide was piloted with three people who have lived experience of accessing mental health services and/or being LGBTQ+ and was amended accordingly. The topic guide was also edited during data collection to better reflect key issues as they emerged from the data. Participants were asked to share their experiences of mental health and accessing mental health services during COVID-19, and to reflect on how their LGBTQ+ identity may have influenced their experiences. Interviews were audio-recorded, transcribed verbatim by HL, anonymised and imported into NVivo 12 [44] for data analysis. Field notes were also taken by HL during and after the interviews to supplement the transcripts and capture immediate reflections. At the end of the interview, participants were provided with a debrief sheet and a £25 online shopping voucher for taking part.

### Data analysis

Reflexive thematic analysis was used to analyse the qualitative data, following the six key stages as outlined by Braun and Clarke [45]; familiarisation, data coding, initial theme generation, theme development and review, theme refinement, and writing up. Reflexive thematic analysis is a flexible approach to making sense of participants’ lived experiences by identifying patterns of meaning within the qualitative data [45]. Specifically, a hybrid approach to thematic analysis was adopted in this study [46], as it incorporated a data-driven inductive approach in coding and theme generation, and a deductive a priori template of codes from Levesque’s Conceptual Framework for Healthcare Access [41]; mental health needs, perception of mental health needs and desire for care, mental healthcare seeking, mental healthcare reaching, mental healthcare utilisation, and mental healthcare consequences (Table 1). The analysis was approached from a critical realist perspective [47], by both reflecting on the a priori knowledge and theories that the research team brought to the analysis, and by enabling the formulation of new knowledge which did not necessarily fit within existing theories. This approach was deemed appropriate as whilst there is a need to undertake theory-driven research in this area [48], there is also a considerable absence of research on LGBTQ+ experiences of accessing mental health services during COVID-19 to be informed by.

**Table 1:** Levesque’s Conceptual Framework for Healthcare Access domains and their descriptions used as a template of codes during data analysis

Name of framework domain	Description of framework domain
Mental health needs	An individual’s need for mental health services and the mental health conditions and associated symptoms participants reported, alongside impacts of the COVID-19 pandemic on mental health.
Perception of mental health needs and desire for care	An individual’s perception of their need for mental healthcare and their desire to access mental healthcare services; two concepts – the approachability of the healthcare system (can people facing mental health needs identify that services exist, can the services be reached, and will the services have an impact) and an individual’s ability to perceive the need for mental healthcare services (determined by factors such as health literacy).
Mental healthcare seeking	If and how an individual sought mental healthcare services; two concepts – the acceptability of the healthcare system (cultural and social factors which determine the possibility of individuals to accept the services) and an individual’s ability to seek mental healthcare services (personal autonomy and capacity to choose to seek mental healthcare and knowledge about services).
Mental healthcare reaching	If and how an individual reached mental healthcare services; two concepts – the availability and accommodation of the healthcare system (are services actually available, are services accessible both in a physical and timely manner) and an individual’s ability to reach mental health services (personal mobility, availability, knowledge to physically reach services).
Mental healthcare utilisation	If and how an individual has utilised mental healthcare services; two concepts – the affordability of the healthcare system (economic capacity for people to spend resources and time to use services) and an individual’s ability to pay for mental healthcare services (economic capacity to pay for healthcare services).
Mental healthcare consequences	The consequences of utilising mental healthcare services; two concepts – the appropriateness of the healthcare system (the fit between services and needs, and the adequacy, quality, and effectiveness of services) and an individual’s ability to engage with mental healthcare (capacity and motivation to participate, decision making, health literacy, self-efficacy, communication).

HL familiarised herself with the data by transcribing the interview recordings and re-reading the transcripts several times whilst noting key reflections. HL generated initial codes by attaching short phrases to sections of the transcripts that resembled the lived experiences of participants relevant to the research questions (inductive, data-driven approach). These initial codes were then clustered by HL into candidate themes that reflected patterns of lived experiences across participants at a broader level than the initial codes and were categorised against the framework (deductive, theory-driven approach). These candidate themes were iteratively shared and discussed with the wider research team, who offered feedback and shared their perspectives on the data in regular meetings held during data analysis. The themes were reviewed, refined, and renamed where necessary as a result of these discussions.

### Research team and reflexivity

Researcher subjectivity is not something to be removed or controlled within the process of reflexive thematic analysis [45]. Generating knowledge is inherently influenced by the researcher and their experiences, and therefore reflexivity can be used as a resource to reflect on how the researcher shapes the research and their engagement with the data [45]. Each member of the research team in this study wrote a reflexivity statement prior to data analysis, acknowledging their personal and professional experiences, and their expectations of the research findings. HL kept a reflexive diary throughout the study to capture a continuous awareness of her positionality and note any challenges that may have influenced the research.

HL held an “insider” status when undertaking this research [49], as she had personal experiences which closely aligned with those of the study participants. HL declared this “insider” status at the beginning of each interview to put participants at ease and create a safe space to share their experiences. This practice was viewed as particularly important for LGBTQ+ participants, due to the hostile conditions that LGBTQ+ people have been subjected to historically in mental health research and during COVID-19. The wider research team consisted of two individuals with experience of delivering mental health services (AB & FL), and two individuals with lived experience of being LGBTQ+ and accessing mental health services in Lancashire and South Cumbria (KH & NC). Their involvement ensured that a range of perspectives could be considered during analysis and created opportunities to highlight any personal biases and acknowledge researcher subjectivity. A Guidance for Reporting Involvement of Patients and Public (GRIPP2) reporting checklist [50], outlining the public involvement activities conducted as part of this study and their impact is presented in Table S1 in Additional file 2.

## Results

**Fig. 1:**
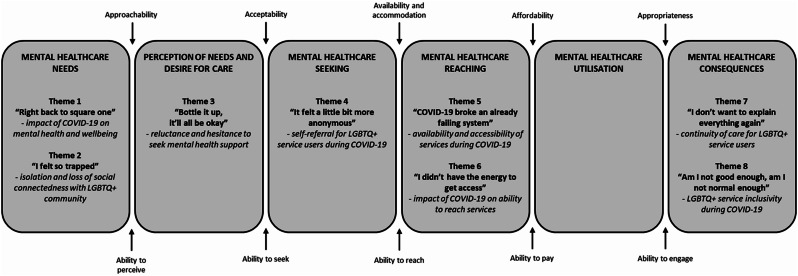
Themes mapped to the domains of Levesque’s Conceptual Framework for Healthcare Access

Twelve participants were recruited for this study. All interviews took place on Microsoft Teams and lasted an average of 59 min (range of 33 to 80 min). Participant characteristics are presented in Table 2. All participants were from a sexual minority group, three identified as a gender minority, and the majority were under 35 years, single, and White British. Participants accessed a range of NHS mental health services during COVID-19, including improving access to psychological therapies (IAPT) services, community mental health teams (CMHTs), and personality disorder (PD) services. Other mental health support was accessed by participants via education, work, and third sector organisations. Eight themes were generated in the analysis, under five domains of Levesque’s Conceptual Framework for Healthcare Access (Fig. 1). The following section provides an in-depth description of the themes, presented with illustrative quotes from participants.

**Table 2:** Summary of participant characteristics

Demographic characteristic		Number of participants (%)
Age group	18–24	2 (17)
	25–34	7 (58)
	35–44	2 (17)
	45–54	1 (8)
Gender	Woman	8 (67)
	Man	2 (17)
	Non-binary	1 (8)
	Gender-fluid	1 (8)
Trans identity	Yes	3 (25)
	No	9 (75)
Sexual orientation	Gay / Lesbian	4 (33)
	Bisexual	4 (33)
	Queer	2 (17)
	Queer / Bisexual	1 (8)
	Asexual / Biromantic	1 (8)
Ethnicity	White – British	10 (83)
	White – Other	1 (8)
	Black / Black British - African	1 (8)
Disability	Yes	8 (67)
	No	3 (25)
	Prefer not to say	1 (8)
Marital status	Single	10 (83)
	Married	1 (8)
	Divorced	1 (8)
Service(s) accessed	IAPT service (NHS)	7 (58)
	Education / Work	4 (33)
	Community mental health team (NHS)	3 (25)
	Third sector (non-LGBTQ+ specific)	3 (25)
	Crisis line (NHS)	2 (17)
	Personality disorder service (NHS)	2 (17)
	Private sector	2 (17)
	Third sector (LGBTQ+ specific)	2 (17)

### Thematic overview

#### Theme 1: “Right back to square one”

All participants had experienced mental health conditions pre-pandemic, from depression and anxiety, through to bipolar and personality disorders. The pandemic had significant impacts on mental health and well-being as it either exacerbated existing mental health conditions or set back any progress that had been made pre-pandemic. Initially the novelty of lockdown meant that adapting to the situation at the beginning felt relatively easy, with some participants finding it a welcome relief from the pressures of normal daily life. However, as time passed, coping with the ongoing pandemic and associated restrictions became more difficult and their mental health deteriorated as a result. The unpredictability of the pandemic, fear of the virus itself, and loss of control, caused significant distress for participants, particularly those with a diagnosis of anxiety.

*“in the early part of the COVID-19 lockdown I felt like I was actually coping alright*,* it was very much one of those things where I felt like I was coping all right and it only later became apparent that I was doing worse than I thought I was” [participant 12]*

*“COVID really obviously exacerbated that*,* and I felt like I couldn’t think more than a couple of weeks ahead*,* that was a big thing for me as well*,* like being able to sort of imagine the future because I didn’t*,* I just couldn’t see” [participant 8]*

Most participants felt that their LGBTQ+ identity wasn’t relevant to their mental health and so hadn’t considered the impact of this during COVID-19. Three participants shared how the pandemic restrictions had provided them with space to undertake some self-reflection about their LGBTQ+ identity. One bisexual cisgender participant found the solitary time helped them become more confident in their LGBTQ+ identity, whilst two queer cisgender participants struggled with internalised homophobia and biphobia as a result of being shut away from the world. Interacting with negative social media about LGBTQ+ people being blamed for COVID-19 had a detrimental impact on how one bisexual cisgender participant was feeling about their identity at the time.

*“I think I became more confident with my sexuality because it actually led me to know who I was a little bit more” [participant 10]*

*“that [social media] was really confronting […] to see that people were so quick to look for someone to lay blame on for something that’s happening all over the world […] it’s gay people’s fault or it’s trans people’s fault […] I’m not interested in hearing my identity attacked just because you don’t want to wear a mask” [participant 5]*

#### Theme 2: “I felt so trapped”

All participants specifically referred to experiencing isolation during the pandemic and the detrimental impact it had on their mental health. Those who lived alone during COVID-19 felt that before social bubbling was implemented, days would go by without human contact and this loss of social connection with others caused distress. They felt they had been let down because of the lack of consideration for people living alone and the extra burden that the loss of human contact had on them. Two participants highlighted that this was a particular oversight of the impact on LGBTQ+ people given that many people from the LGBTQ+ community may have lived alone during COVID-19.

*“I think I struggled a lot with the social isolation that I suffered*,* I was living on my own and didn’t see anybody […] I literally spent days not talking to anybody and I found it really difficult and it had a massive prolonged effect on my mental health” [participant 10]*

*“there was a big thing about the elderly being on their own which is fair enough*,* but you’ve got to realise that the elderly aren’t the only people that live on their own […] I think a lot more awareness that a lot of LGBT people might actually be living alone” [participant 1]*

Frustrations associated with lockdown restrictions meant that some participants struggled with unsupportive living environments and relationship breakdowns. The pandemic restrictions either stopped or reduced access to social support, and not being able to see or touch friends or family contributed to poor mental health through feeling the strains of isolation and loneliness. Participants lost access to social support from the LGBTQ+ community, with LGBTQ+ support groups being paused or moved online, LGBTQ+ spaces being temporarily closed, and loss of contact with LGBTQ+ friends. This loss of social connectedness with the LGBTQ+ community and with people whom participants could relate to caused isolation and exacerbated mental health symptoms. One gay cisgender participant was eager to get back to environments that were supportive of their LGBTQ+ identity (e.g., Pride events). The pandemic happened at a crucial time socially for two queer cisgender participants as they moved from further to higher education and this affected their ability to explore their LGBTQ+ identity with peers leading to confusion and internalised homophobia and biphobia.

*“we’re quite a queer community […] I suppose social norms are not something we particularly abide by and a lot of people say we always like hold hands and hug*,* and I feel like the loss of that was quite difficult for a lot of us” [participant 3]*

*“I just found that anything to do with sort of LGBTQ sort of went out of the window a little bit […] I don’t know whether people just saw it as less important because of what was going on in the world […] for someone like me who struggles with the social side of it [sexuality]*,* I guess it does make it extremely difficult when the ties that you had are then taken away” [participant 10]*

#### Theme 3: “Bottle it up, it’ll all be okay”

Participants felt reluctant or hesitant to make the first step to seek help and felt they should try to continue to cope on their own. Participants only viewed themselves as eligible for mental healthcare once they realised that their mental health was deteriorating and they could no longer cope on their own. Some participants observed the effects COVID-19 was having on the healthcare system and felt that they didn’t want to be a burden on services. Participants lowered their expectations of services and anticipated being rejected by services or turned away. One participant provided their reasoning as *“people have got it worse than me”*. Some participants shared that they would not have waited so long to access support outside of the pandemic. There was also recognition that mental health was not viewed on the same level as physical health during COVID-19 and that this impacted on their hesitance to seek mental health support. Some participants however, found the pandemic acted as a catalyst for them recognising their need for mental health support and challenged their beliefs that they were coping well without support pre-pandemic.

*“I’d always thought that maybe I could be lucky and avoid having to formally access support […] I guess it took the pandemic to kind of disabuse me of that notion” [participant 12]*

Feelings of low self-worth and shame, alongside fears of being judged by services, represented significant barriers to participants’ desire for care and help-seeking behaviour. Some of which were associated with their LGBTQ+ identity.

*“I felt then and sometimes do now a lot of shame about not being able to cope by myself” [participant 5]*

*“I felt like it made me feel like another*,* just like another mentally unwell gay person […] it makes you feel a bit like a cliché maybe” [participant 8]*

*“I think having to disclose those things to people in itself is so daunting*,* it puts you off wanting to access full stop […] you don’t know how they’re going to react” [participant 11]*

#### Theme 4: “It felt a little bit more anonymous”

Many participants had completed a self-referral for IAPT services to access mental health support during COVID-19, with some being signposted to self-refer after consultation with their GP. Self-referral worked better for some participants, offering reasons around self-referral feeling more anonymous, meant they could disclose their LGBTQ+ identity prior to meeting a therapist, and self-referral was validating for their mental health. A bisexual cisgender participant shared how they were ashamed about how they were feeling and that self-referral offered them an opportunity to maintain some anonymity in asking for help.

*“self-referral would have been very good as an option […] if it’s like over the phone or online to kind of do a referral with someone else*,* I feel like there’s a possibility that some things might either get filled in wrong or misinterpreted” [participant 7]*

Some participants however, found the experience of self-referral to be impersonal and they would have preferred a conversation with a healthcare professional to access services rather than fill out a form. One queer cisgender participant felt fobbed off by their GP with a self-referral, and another queer cisgender participant was concerned that without a healthcare professional’s support, a self-referral might be turned down or that they felt like they were making it up.

“that questionnaire made me feel like s**t […] I think it really it forced me to confront like how bad I felt about things” [participant 8]

#### Theme 5: “COVID-19 broke an already failing system”

Most participants had accessed mental health services pre-pandemic and found them to be poor. Those who were already in services as of the start of the pandemic reported that their support either stopped entirely temporarily, the format of their support changed (e.g., moved to remote delivery), or the restart of their support was delayed. Participants who were familiar with services shared that once you’re in services it can feel like you’re getting somewhere, but that initial period of seeking support felt like going around in circles and COVID-19 made this worse. IAPT support was perceived as limited, in that it only included a short series of sessions which did not enable participants to build a relationship with the therapist. Waiting times and delays to access during COVID-19 and the impact they had on their deteriorating mental health were highlighted by nearly all participants. One gay genderfluid participant suggested that interim support for people on the waiting list could have really made an impact during COVID-19 on the hope that they held around getting support in the future. Most participants shared their perspectives about mental health services being under-resourced and under-funded, and appreciated that the pandemic was a challenging time.

*“that’s when they refer me to someone else and then I’ll get referred on to someone else*,* I just end up going in a big circle […] it [the pandemic] made the circle a lot longer with a lot more gaps in it because I was waiting for months” [participant 3]*

*“after about the third or fourth time on the merry-go-round*,* you did start to think well I’m trying to access help but help doesn’t seem to want to help me […] it felt like it [the pandemic] made a system that was creaking a lot worse” [participant 12]*

#### Theme 6: “I didn’t have energy to get access”

Participants felt that they did not possess sufficient resources to access mental health services. They did not feel well enough to engage with support or feel that they had the energy to be persistent and advocate for themselves. Participants were having to fight harder at a time when they had less resources (e.g., social support) and were more in need of the support.

*“I think the reason why I wasn’t recovering was because I didn’t have the energy to be persistent to get the access to the services that I needed […] I didn’t really want to help myself*,* so I needed like a service to kind of come in and step in” [participant 6]*

A lack of knowledge and awareness of available services during COVID-19 and navigating how to go about gaining access to them acted as key barriers to seeking support. Whilst this was particularly pertinent during COVID-19, this was not a new challenge for many participants who also found navigating access pre-pandemic difficult. One bisexual cisgender participant shared their frustration about feeling like they were not taken seriously by services.

*“by like the fifth or sixth time and sort of thinking*,* am I saying the wrong words*,* do I need to say different words in order to be taken more seriously” [participant 12]*

An inability to reach NHS mental health services during COVID-19 caused many participants to consider seeking support from private healthcare providers, but cost was reported as a barrier to this. Other resources that participants associated with accessing mental health services included anonymity and privacy. Almost all of the support received by participants during the pandemic was delivered in a remote format (e.g., telephone, video). Some participants enjoyed the anonymity and flexibility of remote delivery, whilst others struggled with being in unsupportive environments and fear of being overheard discussing their LGBTQ+ identity and/or mental health difficulties. A participant’s openness with their LGBTQ+ identity seemed to influence this preference.

*“something about that anonymity […] I actually think that this iteration during the pandemic of everything being arms length phone calls rather than even video calls*,* that worked really well for me because then I didn’t have to manage someone else’s feelings about how I looked and how I presented” [participant 5]*

*“there were some aspects of my LGBT identity particularly my gender that I was not comfortable having my family know about […] when I was asked questions […] I did feel very restricted on how I could answer because I was worried that someone might overhear*,* I was worried that someone might walk in*,* and that was something that you didn’t get when you had that like face to face appointment” [participant 7]*

#### Theme 7: “I don’t want to explain everything again”

Continuity of care was particularly important, and this was somewhat neglected during COVID-19. Participants had to repeatedly go over their mental health difficulties and relive the experience of sharing this with different professionals. It was also difficult to share their LGBTQ+ identity over and over again, and not having the opportunity to build a relationship with a professional who knew what they needed to know about them in the short timeframe. This lack of continuity of care worsened participants’ mental health and influenced the extent to which they wished to continue to engage with services.

*“now I need to start to get to know somebody else and that just seems like a lot of effort at the moment and then have to start explaining it all again and I don’t want to explain everything again because that just gets me more upset” [participant 1]*

*“it does make a big difference*,* it’s just nice to know that you’re getting to know someone who’s getting to know you*,* it’s not seeing a different person every week […] I think just having someone who knows you on a personal level is just really beneficial […] someone being able to work more therapeutically with you if they understand you a lot better” [participant 10]*

#### Theme 8: “Am I not good enough, am I not normal enough”

Some participants reported receiving neutral responses to disclosure of their LGBTQ+ identity during COVID-19 and did not think it influenced their experiences. Others reported experiences of discrimination from staff, felt staff had a significant lack of awareness of LGBTQ+ issues, and thought that services were lacking in their inclusivity of LGBTQ+ service users.

*“it was extremely difficult as someone who struggles with their mental health and their sexuality to be able to turn around and feel like a valid person in the middle of all that” [participant 10]*

A gay/lesbian genderfluid participant requested to change therapist during their contact with mental health services as they felt that once they had disclosed their sexuality, the therapeutic space no longer felt safe for them.

*“this person doesn’t think that you deserve to access this service […] yeah it did feel very much like do I have to go somewhere else*,* am I not good enough*,* am I not normal enough” [participant 7]*

An asexual biromantic cisgender participant found that their sexuality directly influenced their mental health treatment and was offered sex therapy rather than therapy for the mental health difficulties they were experiencing.

*“when you say you’re asexual*,* people immediately go you’re broken*,* we need to fix this and they focus on that rather than what I’m actually there for*,* which is mental health issues that are completely unrelated to my sexuality” [participant 3]*

Participants recommended a number of ways to improve LGBTQ+ service inclusivity, including more visibility about LGBTQ+ inclusivity (e.g., wearing rainbow badges, staff using pronouns), improving awareness of LGBTQ+ issues among staff, staff training on supporting LGBTQ+ people, challenging cis-heteronormative assumptions of staff, and employing LGBTQ+ professionals who participants thought would be easier to relate to and more validating.

*“I just felt like I could be more open with her […] wanting someone who sort of fits into similar categories to you because it helps have that relatability factor and you maybe think they can understand what’s going on with you” [participant 9]*

*“acknowledge that LGBTQ people have this extra layer of things to consider and level of safety that they may not know […] it’s being very*,* very explicit and clear that it’s a welcome*,* everybody’s welcome*,* and to do things about pronouns and stuff like that” [participant 4]*

## Discussion

### Summary of key findings

This study identified eight themes, under five domains of Levesque’s Conceptual Framework for Healthcare Access [41], describing the experiences of LGBTQ+ people who accessed or tried to access NHS mental health services during the COVID-19 pandemic. The findings suggest that LGBTQ+ people experienced significant disruptions to their mental health and access to mental health services during COVID-19. The findings also convey a sense that LGBTQ+ people have been adversely affected by the pandemic and associated restrictions through experiences of isolation and loss of social connectedness, and challenges associated with having sufficient resources to access mental health services. Participants felt let down both by a lack of support for LGBTQ+ people and a lack of support for mental health during a global crisis preoccupied with physical health. Whilst direct discriminatory experiences were rare, there was an overall perception that services lacked inclusivity and more could be done by services to improve LGBTQ+ people’s experiences of accessing mental health services. The study findings are discussed below in the context of existing literature.

### Findings in the context of existing literature

The findings of this study support evidence that COVID-19 had detrimental impacts on the mental health of LGBTQ+ people [19, 20]. All participants reported mental health conditions that pre-dated the pandemic, which were exacerbated by the restrictions implemented to control the spread of the virus. These findings offer support for minority stress theory [6], with mental ill health being the consequence of cumulative exposure to distal and proximal stressors pre-pandemic and then during the pandemic. Gillard et al. [51] also found that people with pre-existing mental health conditions were adversely impacted by the pandemic as they were starting from a lower level of psychological well-being and social connection. Kneale and Bécares [23] highlighted that COVID-19 magnified systems of oppression and coincided with intensifying hostile attitudes about sexuality and gender identity on a global scale, which in turn partially explained the increased vulnerabilities to depression and perceived stress for LGBTQ+ people during COVID-19. Most participants did not view their LGBTQ+ identity as relevant to their mental health, which could be associated with internalised stigma, in that they have absorbed negative stereotypes around being LGBTQ+ [6] and mental ill health [52], and as a result of these cumulative forms of discrimination felt the need to reiterate that the two concepts are separate. In the context of the current study, the intersectional effect of being LGBTQ+ and having a pre-existing mental health condition may have created conditions during the pandemic that exacerbated mental distress and poor well-being for these population groups. Minority stress and as such the absence of social safety (e.g., inclusion, belonging, protection) creates and maintains health disparities for stigmatised groups such as LGBTQ+ people [9], which is likely to have been amplified during pandemic conditions. Therefore, in times of crisis such as a pandemic, policy makers need to go beyond just identifying who is vulnerable and consider how existing societal structures (e.g., cis-heteronormativity) exacerbate inequalities and not only maintain vulnerabilities but produce them [53]. Applying an intersectional approach would be essential in this instance for creating mental health services that effectively acknowledge and address the diverse needs of LGBTQ+ people.

Isolation and loss of social connectedness with the LGBTQ+ community during COVID-19 caused significant distress for participants. Whilst this is not surprising given the pandemic caused isolation across the general population, existing evidence suggests that this experience may have been more significant for LGBTQ+ people [20, 54, 55]. One of the reasons for this may be that social support systems for LGBTQ+ people are more likely to exist outside of the home [56], with older LGBTQ+ people more likely to live alone and younger LGBTQ+ people more likely to live with unsupportive family. In the context of COVID-19, access to “chosen family”, a concept highlighting how LGBTQ+ people rely on friendship networks to compensate for a lack of familial support [56], was more likely to be restricted and as such protection against isolation for LGBTQ+ people was less likely. The present study’s findings suggest that existing mental health inequalities for LGBTQ+ people were compounded by pandemic restrictions through isolation and loss of social connectedness. Social connectedness with the LGBTQ+ community offers protection against stress and loneliness through the mechanism of reducing the impacts of marginalisation [57], but during the pandemic opportunities to connect with the LGBTQ+ community were diminished. As such a major protective buffer against mental ill health for these population groups was significantly reduced.

Participants shared a reluctance and hesitance to seek mental health support during COVID-19. LGBTQ+ people with mental health conditions experience a double stigma as the stigma associated with being LGBTQ+ intersects with that associated with having a mental health condition [52]. Stigma is a well-documented barrier to help-seeking behaviour [52], and could potentially explain why participants in this study were reluctant or hesitant to seek help for their mental health. As a result of exposure to distal stressors (e.g., discrimination, prejudice), some participants reported experiencing proximal stressors, such as feelings of low self-worth and shame, and fears of being judged, which in previous evidence has been associated with unwillingness to seek support from mental health services [58]. Perceived stigma, that which is created by the expectations of being stigmatised, is associated with low self-esteem and wishing to avoid events that have a high chance of experiencing rejection [59]. The Candidacy framework describes how people assess their eligibility for care and suggests that vulnerable groups, such as LGBTQ+ people, may have a different identification of candidacy in that they are more likely to seek help at crisis point rather than earlier and put off seeking help initially in fear of being judged by professionals [42]. Liberati et al. [60] found that the pandemic had significant impacts on the identification of candidacy for people with pre-existing mental health conditions, with service users reporting uncertainty about the level of distress that needed support, questioning whether they deserved access to support, and a low sense of self-worth accompanied by perceptions of being burdensome. Many of these experiences resonate with those shared by participants in the current study, however these were compounded by thoughts around ineligibility for mental healthcare as a result of issues associated with their LGBTQ+ identity (e.g., low self-worth, shame, fear of being judged).

Self-referral facilitated help-seeking for some participants during COVID-19 as it enabled a sense of anonymity which couldn’t be achieved through typical access routes (e.g., GP). A self-referral option was introduced for IAPT services as a potential way to improve accessibility and close the access gap for marginalised groups [59]. Within the context of the pandemic, self-referral for IAPT services was likely implemented at a greater extent due to the inaccessibility of GPs, previously viewed as the gatekeepers of access to psychological interventions. Habicht et al. [61] found that a self-referral chatbot for IAPT services increased overall referrals, but particularly increased referrals for minority groups (e.g., bisexual people, non-binary people), suggesting that the self-referral process may better facilitate help-seeking for minority groups. People from gender minority groups particularly valued the human-free nature of self-referral as it enabled them to seek support without fear of judgement or discrimination [61]. Bisexual and gender minority participants in the present study also indicated a preference for self-referral, providing similar reasons around anonymity and avoiding judgement.

The COVID-19 pandemic had significant effects on the extent to which people could navigate seeking mental health support, and thus created conditions that required a higher degree of resources, commitment, perseverance, and competence to negotiate a point of entry to mental health services [60]. LGBTQ+ people are less likely to possess the resources needed to navigate mental health service access during COVID-19. They have an increased risk of low self-esteem, shame, stigma, and discrimination [58, 62]; all of which impact help-seeking behaviour and beliefs about eligibility for care, and less access to social support that is affirming of their identity, during a hostile time for sexual and gender minorities, to mitigate those risk factors [21]. Findings from the current study suggest that LGBTQ+ people experienced deteriorating mental health from a lack of access and thus were unequally impacted by the pandemic. In addition to navigating a point of entry to services, COVID-19 also caused a dramatic shift in the way mental health services were delivered, where delivering care remotely rapidly became the default option [63]. In the current study, remote delivery was not always appropriate for LGBTQ+ service users as they lacked privacy to engage in the therapeutic space when living in unsupportive environments. On the other hand, remote mental healthcare bypassed the stigma for some participants as they could retain some anonymity and were less concerned about being judged. Whaibeh et al. [64] suggested that telepsychiatry could help to address the mental health treatment gap for LGBTQ+ people by reducing the challenges faced by these population groups (e.g., stigma). Whilst the current study’s findings indicate that offering a preference for LGBTQ+ service users may be of benefit, this should not replace efforts to address the lack of inclusivity within services and delivering care in a sensitive and affirming way to ensure that no LGBTQ+ person feels unsafe to access mental healthcare in person or virtually. Economic capacity wasn’t viewed as a barrier to accessing mental health services, which is likely due to the provision of universal mental healthcare in the UK.

Building a relationship with a therapist was important to participants in this study. Therapeutic alliance is crucial for all service users to engage with mental health treatment and achieve successful outcomes [65]. The relationship between a mental health professional and LGBTQ+ service user has been shown to influence mental health service use for sexual and gender minorities [66]. For LGBTQ+ service users, concealing sexual orientation and/or gender identity indicates poor therapeutic alliance and as such can undermine treatment success [66], which may explain why many participants felt that their therapy did not improve their mental health. A strong therapeutic relationship with LGBTQ+ service users has been found to be strongly correlated with affirmative practices (e.g., professional knowledge and awareness, and cultural competence) [67]. De Moissac et al. [68] state that professionals proactively addressing issues associated with one’s sexual orientation and/or gender identity (e.g., internalised stigma) and focusing on the holistic integration of one’s identity is essential for treatment efficacy. Some participants in the present study shared their preference for a mental health professional who also identified as LGBTQ+ as they are more likely to empathise with their lived experience. This could be potentially unnecessary if therapeutic alliance was effectively achieved through the delivery of affirming mental healthcare.

Continuity of care was also perceived as important by study participants. Repeated disclosure of their LGBTQ+ identity and their mental health difficulties to multiple professionals created discomfort for participants, with many choosing to stop disclosing their identity to services as a result. Continuity of care was specifically challenging during COVID-19 as mental health services had to reorganise and work within structures that weren’t feasible in their usual practice. Continuity of care in mental health services increases trust, service user satisfaction, and disclosure of information, and can contribute to better treatment outcomes [69]. The fragmentation of services and care discontinuity experienced by participants during COVID-19 is likely to have affected all service users, but could have had potentially disproportionate effects for LGBTQ+ people as they are less likely to trust healthcare professionals and so may not have disclosed information [12], and thus treatment may have been less effective. This also feeds into explaining why participants felt that the support they accessed during the pandemic did not improve their mental health.

Having mental health professionals and services that are able to deliver care that is inclusive, sensitive, and affirming for LGBTQ+ people is critical to enhancing the effectiveness of mental health treatment, addressing mental health inequalities, and reducing risk of disengagement [11]. A lack of knowledge and training on supporting LGBTQ+ people can perpetuate stigma through the maintenance of cis-heteronormative assumptions [11]. A lack of LGBTQ+ service inclusivity was conveyed by participants as they felt that professionals needed better knowledge and training to work with LGBTQ+ people and that this would ultimately improve their experience of accessing services. Participants proposed a range of ways to improve LGBTQ+ service inclusivity (e.g., visibility, staff training, rainbow badges, pronoun use), many of which are reflected in the Health and Care LGBTQ+ Inclusion Framework published in 2022 [70]. With a continued loss of focus on improving the health of LGBTQ+ people, progress has been slow in implementing these recommendations in England, and therefore services continue to be perceived as non-inclusive. In the United States of America (USA), Fish et al. [11] established a “Sexual and Gender Diverse Learning Community” during COVID-19 to improve the mental health workforce’s competence in supporting LGBTQ+ service users and found the programme’s implementation to be both acceptable and feasible. Similar programmes are needed in England to assess the effects of implementing recommendations to improve LGBTQ+ service users’ experiences of mental health services.

### Strengths and limitations

This study had a number of strengths and limitations. Few studies have conducted interviews with LGBTQ+ people about their experiences of mental health and accessing mental health services during COVID-19 in the UK, and most have relied on online surveys. The UK saw unprecedented disruptions to the delivery of healthcare services during the pandemic and this happened amidst existing disparities for LGBTQ+ people, therefore offering an opportunity to gain insight to ensure a more equitable response in the event of future crises. This study captured in-depth experiences of population groups who are often seldom heard within health research and has corroborated findings from survey studies [21–23, 71]. The perspectives of people with lived experience of accessing services and being LGBTQ+, and of people with professional experience of delivering mental health support were embedded into this research at every stage from design, undertaking, through to analysis and interpretation. This meaningful stakeholder involvement enhanced reflexive practice and the validity of the findings. The researchers felt that declaring an insider perspective during interviews helped to put participants at ease and as a result, participants’ accounts may have been more open and honest. One participant reflected on how refreshing it was for LGBTQ+ research *“being done by us rather than to us”*. A systematic analysis method was adopted in this study, alongside the use of an established framework of healthcare access to code the data. Whilst this approach may have helped to situate the findings within broader literature and helped to consider the complexity of access, it may have led to the oversimplification of concepts. This study captured experiences from a range of LGBTQ+ identities, with different mental health conditions, who accessed a range of mental health services in Lancashire and South Cumbria.

Despite efforts to recruit through LGBTQ+ networks and reach digitally excluded participants through the use of paper posters in the local area, recruitment for this study was challenging. Due to the time restraints and these recruitment challenges, the study has a relatively small sample size and was limited to a specific geographical area. A minimum of twelve interviews were conducted in an effort to achieve saturation and understand common experiences amongst LGBTQ+ people, as suggested by previous research [72]. However, a limitation of this research is that the target population of LGBTQ+ people is unlikely to be an homogenous group and it was unable to capture the perspectives of some identities within the LGBTQ+ community (e.g., gay cisgender males, bisexual transgender people), therefore saturation may not have been achieved. Whilst relatively diverse in terms of sexual orientation and gender, the sample lacked diversity in age, ethnicity, and marital status, and therefore may not have captured the views of older people, ethnic minorities, or married people. This study therefore is unable to capture the nuances of how these intersecting identities may have influenced the experiences of accessing mental health services during COVID-19. Minority population groups, such as LGBTQ+ people, are under-represented in health research and are difficult to recruit, often due to a lack of trust [73]. To further address this issue, the insider perspective could have been declared on the study adverts to engender a sense of trust with potential participants from the outset. Lastly, the interviews were conducted two to four years after the experiences being explored. Some participants had difficulties recalling some details which is not surprising given the impact the pandemic and the length of time passed, and therefore this may have influenced participants’ recollection of what happened. On the other hand, this may have also given participants time to reflect and make sense of their experiences.

### Implications for practice

This study raises important implications for mental healthcare practices associated with supporting LGBTQ+ people experiencing mental ill health. In the event of significant disruptions to services (e.g., crisis conditions), maximising protective factors (e.g., social support, social connectedness with the LGBTQ+ community) and enabling timely access to effective mental health support is necessary to mitigate against the adverse effects identified in this study for LGBTQ+ people. Stigma-reducing interventions for LGBTQ+ people who have mental health conditions could be considered to address barriers to help-seeking. Mental health services need to specifically adapt to address barriers to reaching mental health support for LGBTQ+ people. Adopting flexibility where possible in offering referral methods and treatment delivery options and preferences to improve equity of access for LGBTQ+ people was suggested as a potential solution by participants.

LGBTQ+ service users did not perceive mental health services as inclusive during COVID-19. Whilst reassuring that there were limited direct discriminatory experiences, services need to address this lack of inclusivity. A range of recommendations that services could implement were suggested by participants to ensure that services provide mental healthcare that is sensitive and affirming for LGBTQ+ people; actions which should not be neglected under pandemic or crisis conditions given the higher mental health burden for LGBTQ+ population groups. Services should be more visibly LGBTQ+ inclusive from the outset so that LGBTQ+ people feel that they are welcome to access services. Inclusive communications and marketing, staff wearing rainbow badges, and introducing themselves with their pronouns are some of the strategies suggested by participants to improve the experiences of LGBTQ+ people. Rolling out a training programme or learning community with ongoing professional development opportunities to improve the knowledge, awareness, and competencies of professionals supporting LGBTQ+ services users could challenge staff cis-heteronormative assumptions and lead to the meaningful inclusion of affirmative practices in mental healthcare for LGBTQ+ people. Training of a similar nature could also be delivered in the early education of healthcare professionals (e.g., medical and nursing degrees) so that affirmative practices are embedded from the outset. This would enable professionals to acknowledge their biases, challenge their assumptions, and deliver more inclusive mental healthcare which would effectively enhance therapeutic alliance with LGBTQ+ services users and thus increase treatment efficacy. Many of these recommendations are widely reflected in existing best practice guidelines [70, 74–76].

### Implications for research

As LGBTQ+ people continue to demonstrate an increased risk of mental health conditions and are having poor experiences of accessing mental health services, further research is needed to understand the ways in which services can better meet the needs of LGBTQ+ people and assess the impact of these changes. It is important to generate learning about how to mitigate against these inequities in the event of future pandemics or similar events. Capturing the perspectives of mental health professionals working with LGBTQ+ services users in mental health services would be valuable to generate insight of what barriers exist to delivering inclusive and responsive support for LGBTQ+ people and how best to equip them. Ultimately, co-developing a training package or learning community with staff and LGBTQ+ service users, as recommended by this study’s participants, which aims to address the lack of knowledge, awareness, and competencies of professionals working with LGBTQ+ people in mental health services, is a vital next step of research. With this, a comprehensive evaluation of the feasibility and acceptability of rolling out such a programme, and assessing the effectiveness of having more informed and trained staff on LGBTQ+ experiences of accessing mental health services, would be necessary.

## Conclusion

The findings of this study validate earlier evidence that LGBTQ+ people may have been detrimentally affected by the COVID-19 pandemic, through an increased risk of mental ill health, isolation, and loss of social connectedness. This study captured novel findings around the challenges to accessing NHS mental health services during COVID-19, including experiences of stigma and discrimination, concerns about disclosing their LGBTQ+ identity, living in unsupportive environments and being unable to access support delivered remotely, and not possessing certain resources to navigate access. Where positive experiences of access were identified (e.g., offering self-referral options and treatment delivery format choices, continuity of care), these highlighted opportunities for change. In future planning for similar significant events, policy makers should not overlook the potential vulnerabilities of LGBTQ+ population groups and should endeavour to mitigate any detrimental impacts. Beyond pandemic conditions, the inclusivity of mental health services requires improvement, and this paper has outlined a series of implications for both practice and research, which could be considered in order to improve the experiences of LGBTQ+ people accessing NHS mental health services. 

## Supplementary Information

Below is the link to the electronic supplementary material.

Supplementary Material 1

Supplementary Material 2

## Data Availability

The datasets generated and analysed during the current study are not publicly available due to the potential identifiability of participants and the sensitive nature of the research. However, some supporting data is available from the corresponding author upon reasonable request.
