# Supplementary material for: LGBTQ+ experiences of accessing NHS adult mental health services during COVID-19 in an area of North West England: a qualitative interview study
Source: BMC Health Serv Res. 2026 Feb 7;26:354. doi: 10.1186/s12913-026-14014-2 (PMC12977796; doi:10.1186/s12913-026-14014-2)
Supplement: Supplementary file 1 — Supplementary Material 1 [file 12913_2026_14014_MOESM1_ESM.docx]

**Additional file 1**

**Figure S1.** Interview topic guide


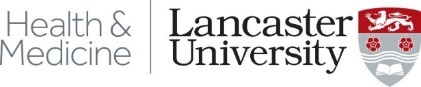

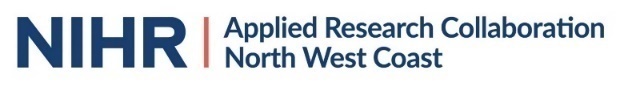


**Interview Guide**

**Title of study: LGBTQ+ experiences of accessing adult mental health services during COVID-19**

**Pre-interview contact to check participant meets eligibility criteria, check receipt of participant information sheet, check completion of consent form and monitoring form**

**Introduction checklist**

**Introduce myself** – PhD student in the Medical School at Lancaster University, pronouns are she/her, part of the LGBTQ+ community (bisexual cis woman)

**Provide a brief overview of the purpose of the interview** – thank for agreeing to take part, interviews are part of a PhD project to understand LGBTQ+ experiences of accessing or trying to access adult mental health services during COVID-19, interview will involve asking you a series of questions about your experiences of mental health during COVID-19, accessing or trying to access services during COVID-19, and being LGBTQ+, the findings will hopefully help to inform changes to mental health services to improve access for LGBTQ+ people and add to the literature of how COVID-19 affected LGBTQ+ people

**Provide a brief description of how the interview data will be used and stored** – the data collected from this interview will by anonymised and stored securely at Lancaster University, will contribute to completion of PhD, will be published in journal articles and doctoral thesis, all data will be destroyed after 10 years, check understanding at this stage and any questions

**State approximately how long the interview will take** – approximately 60 minutes

**Remind participant about confidentiality and its exceptions** – will uphold confidentiality unless there is a risk of harm to you or others, will need to inform academic supervisor in this case, if possible will let you know if need to do this

**Confirm informed consent to participate in the interview** – check you are happy to continue

**Confirm right to withdraw at any point during the interview –** remind that you can withdraw from the interview at any time without providing a reason, you can stop the interview at any time without providing a reason, interview will be discussing sensitive issues and experiences, you do not have to answer any questions you do not want to answer

**Confirm consent for the interview to be recorded (start recording at this stage)** – check you are happy to be recorded (if participant does not consent confirm consent, PhD student to takes notes during the interview), switch on recording and just re-confirm you are happy to be recorded whilst recording

**Ask if participant has any questions before the interview starts**

**Interview questions**

1. **Perception of mental health needs and desire for care** *(experience of need for mental healthcare)*

- Can you tell me about how your mental health was during the COVID-19 pandemic (*between March 2020 and February 2022*).
  - *Prompts:* How did this differ to pre-pandemic (*before March 2020*)? Did the pandemic and associated restrictions (*e.g., lockdowns*) specifically affect your experience of mental health, if so how?
  - *Prompts:* What help did you feel you needed at this time? What sort of help were you hoping for? What help did you access?

1. **Healthcare seeking** *(experience of seeking mental healthcare)*

- Can you tell me about your experience of seeking help for your mental health during the pandemic.
  - *Prompts:* What did you do? Where did you go first for help with your mental health (*e.g., GP, A&E, LGBTQ+ organisation, direct to mental health services, online resources*)? Why did you go here first? How did you contact them?
  - *Prompts:* Did the pandemic affect your choice in where you went to for help, if so how? If you have sought help before, how did your choice differ from pre-pandemic?
  - *Prompts:* Did your LGBTQ+ identity affect your choice in where you went to for help, if so how?
  - *Prompts:* Was there anything else that affected your choice in where you went to for help?

1. **Healthcare reaching** *(experience of gaining or not gaining access to mental health services)*

- Can you tell me about what happened after seeking help for your mental health during the pandemic.
  - *Prompts:* What was it like when you tried to get help for your mental health?
  - *Prompts:* Were you referred to mental health services (*e.g., IAPT, CMHTs, CRHTs EIS*)? What contact did you receive from this service/s? What form did this contact take (e.g., face-to-face, telephone, video)?
  - *Prompts:* Did the pandemic impact on what happened after seeking help for your mental health, if so how?
  - *Prompts:* Did your LGBTQ+ identity impact what happened after seeking help for your mental health, if so how? During contact with services, were you asked about your sexual orientation and/or gender identity? If so, what was your experience of this?
  - *Prompts:* Was there anything else you feel impacted what happened after seeking help for your mental health?

1. **Healthcare utilisation** *(experience of using mental health services once accepted)*

- (*if referred to/accepted by mental health services*) Can you tell me about your experience of using mental health services during the pandemic.
  - *Prompts:* Did you have regular contact with the service/s? What form did this contact take (e.g., face-to-face, telephone, video)?
  - *Prompts:* Were you already in contact with this service or other mental health services before the pandemic? If so, how did your experience differ before and during the pandemic?
  - *Prompts:* Did the pandemic affect how you used this service/s, if so how?
  - *Prompts:* Did your LGBTQ+ identity affect how you used this service/s, if so how? Were you asked about your sexual orientation and/or gender identity? If so, what was your experience of this?
  - *Prompts:* Was there anything else you feel impacted how you used mental health services?
- (*if not referred to/accepted by mental health services*) Did you use any other sources of support during the pandemic (*e.g., LGBTQ+ organisations, third sector organisations, online forums, friends, and family*)? What were these sources of support like?

1. **Healthcare consequences** *(outcomes and satisfaction with mental health services)*

- Can you tell me about your overall experience of accessing or trying to access mental health services during the pandemic.
  - *Prompts:* Were you satisfied with the contact you had with services? How did it help (*or not help*) your mental health? Were there any particularly positive or negative experiences?
  - *Prompts:* Are there any changes you would recommend to improve access to services from your experience of using mental health services during a pandemic? Are there any changes you would recommend to improve access specifically for LGBTQ+ people?
  - *Prompts:* If you had to access services again (*either during a pandemic or not*), would you do anything differently next time? If so, what?

1. **Other;**

- Is there anything further we haven’t talked about that you would to add?

**Debrief checklist**

**Thank for participation in the interview and summarise the next steps (stop recording at this stage)** – the recording of the interview will now be transcribed and any identifiable data will be anonymised, all stored securely and deleted after 10 years, data will be combined with other participants’ responses and analysed, findings will be written up and published in journal articles and doctoral thesis

**Provide the participant with the £25 shopping voucher** – send via email if interview conducted remotely (check receipt), or give in person if interview conducted face-to-face

**Confirm the participant is happy to be contacted after the interview if necessary** – check what method

**Ensure the participant is happy with the interview and there are no safeguarding/well-being concerns**

**Provide the participant with the debrief sheet** – highlighting the resources at the end of the sheet and the relevant contact details if the participant has any further questions or queries
