# Supplementary material for: LGBTQ+ experiences of accessing NHS adult mental health services during COVID-19 in an area of North West England: a qualitative interview study
Source: BMC Health Serv Res. 2026 Feb 7;26:354. doi: 10.1186/s12913-026-14014-2 (PMC12977796; doi:10.1186/s12913-026-14014-2)
Supplement: Supplementary file 2 — Supplementary Material 2 [file 12913_2026_14014_MOESM2_ESM.docx]

**Additional file 2**

**Table S1.** GRIPP2 reporting checklist describing the public involvement conducted as part of this research

| **Section and topic** | **Details** |
| --- | --- |
| Aims and objectives of the PPI | The PPI activities for this study ultimately aimed to ensure that the research was appropriate and sensitive to the context of LGBTQ+ mental health, and to enhance the validity and applicability of the research findings. The objectives of the PPI activities were specifically to involve public advisers in;   - defining research questions that were relevant and a priority to them, - designing the research to ensure methods were ethical and appropriate, - reviewing study documentation to improve the accessibility and inclusivity of participant materials, - identifying participant recruitment barriers and ways to overcome them, - the analysis and interpretation of research findings, - and the development and dissemination of outputs to share research findings. |
| Methods used for PPI | The PPI activities conducted as part of this study included;   - recruiting two public advisers with lived experience of being LGBTQ+ and accessing mental health services in the area, - presenting initial research ideas and questions to a public adviser forum and the NHS Trust service user research group for their review and feedback, - conducting three virtual meetings using Microsoft Teams with a public adviser to discuss the research questions, study design, and participant recruitment, - sharing participant documentation via email with two public advisers for their review and feedback, - conducting pilot interviews using Microsoft Teams with two public advisers for feedback on the interview guide and interviewing technique, - conducting four hybrid meetings attended either virtually on Microsoft Teams or in-person with two public advisers to share preliminary findings and discuss their interpretation, - and sharing a draft of the journal article via email with two public advisers for their review and including them as co-authors on the final version. |
| Results of PPI | The PPI activities took place across different stages of the research and contributed to how the research was conducted in a variety of ways;   - *Research questions:* Public advisers and NHS Trust service users confirmed the importance and relevance of the proposed research and questions from a lived experience perspective, particularly highlighting the need for the research in this geographical area. They suggested refining the research questions to not only consider access as a single time point but to also consider the complex journeys service users often experience when accessing mental health services. As such, Levesque’s Conceptual Framework for Healthcare Access was embedded into the research to enable the conceptualisation of access as a complex concept. - *Study design*: Public advisers and NHS Trust service users suggested that interviews would be more appropriate for LGBTQ+ participants due to the sensitive nature of the research and would enable participants to be more open than alternative methods such as focus groups or surveys. They also suggested that giving participants the option of having the interview face-to-face or virtual would also be beneficial to improve participant experience, and did not believe that offering a £25 voucher as reimbursement for taking part was an undue inducement. The design of this study was subsequently informed by these recommendations. - *Participant recruitment*: A public adviser recommended expanding the LGBTQ+ initialism in the qualitative study advert to help potential participants identify themselves as eligible to take part. The advert was amended accordingly. A public adviser suggested putting up paper copies of the study advert rather than just relying on electronic methods (e.g., social media) to avoid digital exclusion of some participants and also recommended some additional LGBTQ+ specific spaces to potentially recruit participants from. Paper copies of the study adverts were placed in various cafes and LGBTQ+ specific spaces in areas local to the researchers, which may have contributed to the recruitment of four additional participants. - *Data collection*: Two public advisers reviewed participant documentation study and recommended changes to some of the wording used across the documents to improve accessibility and inclusivity (e.g., writing LGBTQ+ out in full so that potential participants felt included, adding that taking part will not affect the care participants receive from mental health services). Two public advisers took part in a pilot interview and recommended changes to the interview guide to reduce repetition and offer more opportunities to prompt participants to expand (e.g., using phrases such as “what was it like”, not being too specific about the services accessed to allow participants to use their own terminology). The participant documentation and interview guide were amended as a result of the feedback from public advisers. - *Analysis and interpretation*: Two public advisers were included in the wider research team which met four times during the data analysis stage. Emerging findings were shared iteratively with the group to ensure that the researchers had appropriately interpreted the meaning of participants’ experiences. Public advisers shared their thoughts on the disproportionate impacts the pandemic may have had on the mental health of LGBTQ+ people and their access to mental health services, and reflected on how these resonated with the patterns emerging from the interview data; all of which added validity to the findings. - *Dissemination*: Two public advisers have been included in the authorship of this journal article and provided feedback on the final draft, again suggesting a few changes to the language to ensure its accessibility for lay audiences. |
| Discussion and conclusion of the PPI | The knowledge and experience of public advisers and service users throughout this research was effective in helping to contextualise the complexities of accessing mental health services and the potential challenges LGBTQ+ people experience, during and beyond the COVID-19 pandemic. PPI activities were particularly supportive in ensuring that the research was conducted in a sensitive and inclusive manner, and raising issues that had not been considered by the researchers. The researchers found conducting pilot interviews with public advisers extremely valuable prior to data collection to practice professional interviewing skills. A number of changes were made to the research as a result of the input from public advisers and service users, which ultimately influenced not only the validity and applicability of the research findings, but also the practical undertaking of the research (e.g., participant recruitment, interviewing technique). In addition, the researchers endeavoured throughout the research to feed back to public advisers how their involvement had influenced the studies. |
| Reflections/critical perspective of the PPI | The experience of working collaboratively with public advisers was overall positive and the researchers found it both supportive and rewarding. There were some challenges in embedding PPI activities into the timeline of the research (e.g., time constraints, engagement issues), which meant that the impact of PPI may not have always been maximised. For example, it was sometimes difficult to get a timely response from public advisers to arrange follow-up meetings or request feedback on documents, and so their views were not always adequately incorporated into the research. Both of the public advisers identified as sexual minority cisgender males. As a result, there was no inclusion of a gender minority voice in the research despite efforts to recruit via various channels. Public advisers were asked for their views on how the PPI was conducted during the research; both highlighted that the researchers were receptive to feedback and that they found it to be an interesting experience. |
